# Critical Circulatory Failure Accompanied With the aVR Sign Caused by Severe Graft Kinking Related to Acute Pseudoaneurysm Formation After Type A Acute Aortic Dissection Repair

**DOI:** 10.7759/cureus.58027

**Published:** 2024-04-11

**Authors:** Yuta Inoue, Shohei Mitta, Yukihiro Matsuno, Yukio Umeda

**Affiliations:** 1 Department of Cardiovascular and Thoracic Surgery, Gifu Prefectural General Medical Center, Gifu, JPN

**Keywords:** va-ecmo, avr sign, pseudoaneurysm, graft kinking, critical circulatory failure

## Abstract

The aVR sign characterized by ST-segment elevation in lead aVR and diffuse ST-segment depression on the electrocardiogram indicates potential life-threatening conditions. We report the case of a 53-year-old male with a history of ascending aortic replacement for acute aortic dissection, who presented to our institution in shock. The initial electrocardiogram revealed the aVR sign, consisting of ST-segment elevation in lead aVR and ST-segment depression in leads II, III, aVF, and V3-6, leading to the initiation of salvage veno-arterial extracorporeal membrane oxygenation (ECMO) due to deteriorating hemodynamics. The aVR sign resolved shortly after ECMO initiation, and hemodynamics stabilized even with reduced ECMO flow. Subsequent coronary angiography showed no impaired coronary perfusion, whereas contrast-enhanced CT revealed severe supra-valvular stenosis due to pseudoaneurysm-induced graft kinking. The patient was then managed with emergency surgery for the pseudoaneurysm. In this report, we encountered a salvaged case of critical circulatory failure presenting with the aVR sign due to severe graft kinking caused by pseudoaneurysm formation.

## Introduction

The electrocardiogram (ECG) finding characterized by ST-segment elevation in lead aVR and diffuse ST-segment depression is known as the aVR sign [1]. This sign typically reflects transmural ischemia of the basal part of the interventricular septum due to left main trunk (LMT) obstruction in acute coronary syndrome [2].

However, the aVR sign may also be present in other critical conditions. Takotsubo cardiomyopathy can cause the aVR sign due to multivessel epicardial spasm, diffuse coronary microvascular dysfunction/spasm, or direct catecholamine-mediated cardiomyocyte injury. This results in transient global myocardial ischemia of the basal part of the interventricular septum, leading to the aVR sign [2]. Regarding pulmonary embolism (PE), studies have shown that the presence of an aVR sign is associated with a severe clinical presentation, higher median troponin T levels, centrally located thrombi, and in-hospital mortality [3]. Huang et al. reported a case of aVR sign in severe aortic stenosis (AS), where the aVR sign reflects extensive severe subendocardial ischemia in critical AS and is likely to be fatal without appropriate treatment, such as LMT occlusion and PE [4].

Therefore, the aVR sign is considered to be a fatal indicator. Here, we report a case of critical circulatory failure accompanied with the aVR sign caused by severe graft kinking related to acute pseudoaneurysm formation after repair of type A acute aortic dissection.

## Case presentation

A 53-year-old man was transported to our hospital by emergency medical services for sudden onset of chest pain and blackout. The patient had a history of ascending aortic repair for type A acute aortic dissection eight months prior. Contrast-enhanced CT scan at discharge did not note pseudoaneurysm or graft kinking.

**Figure 1:**
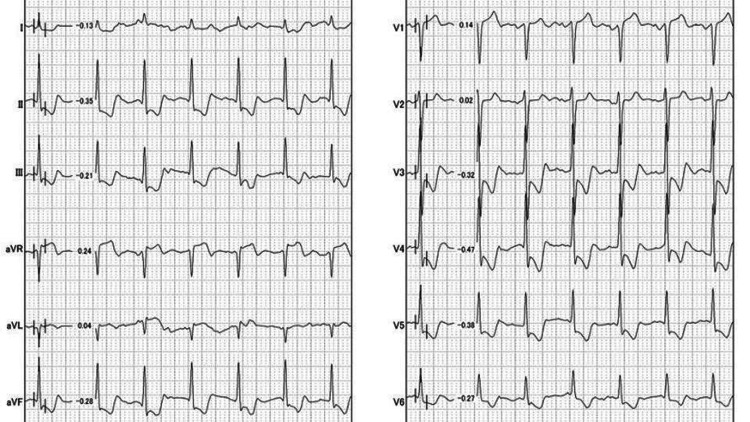
Electrocardiogram on admission. Electrocardiogram on admission showed ST-segment elevation in lead aVR and ST-segment depression in vast leads (II, III, aVF, V3-6).

On his arrival at the emergency department, he was in shock (blood pressure, 60/48 mmHg) with ST-segment elevation in lead aVR and ST-segment depression in vast leads (II, III, aVF, V3-6) on ECG, the so-called aVR sign (Figure 1).

Blood tests on admission showed decreased hemoglobin and elevated lactate dehydrogenase and N-terminal pro-brain natriuretic peptide. Arterial blood gas analysis showed poor oxygenation and elevated lactate levels (Table 1).

**Table 1:** Blood tests and arterial blood gas analysis on admission. Blood tests on admission showed decreased hemoglobin and elevated LDH and NT-proBNP. Troponin I was negative. Arterial blood gas analysis showed poor oxygenation and elevated lactate levels. WBC = white blood cell; RBC = red blood cell; CK-MB = creatine kinase-myoglobin binding; AST = aspartate transaminase; ALT = alanine transaminase; LDH = lactate dehydrogenase; γ-GTP = gamma-glutamyl transferase; BUN = blood urea nitrogen; CRP = C-reactive protein; NT-proBNP= N-terminal pro b-type natriuretic peptide; APTT = activated partial thromboplastin time; PT-INR = prothrombin time-international normalized ratio

Parameter	Result
WBC	6,400/µL
RBC	2.89 × 10^6^/µL
Hemoglobin	8.1 dL
Hematocrit	23.3%
Platelet	31.3 × 10^4^ µL
Total protein	6.7 g/dL
Albumin	4.1 g/dL
Creatinine kinase	185 IU/L
CK-MB	12 IU/L
Total bilirubin	1.85 mg/dL
AST	37 IU/L
ALT	8 IU/L
LDH	706 IU/L
γ-GTP	13 IU/L
Amylase	35 IU/L
Creatinine	1.12 mg/dL
BUN	19 mg/dL
Na	141 mEq/L
K	3.4 mEq/L
Cl	107 mEq/L
Calcium	8.64 mg/dL
CRP	0.12 mg/dL
Blood sugar	145 mg/dL
HbA1c	4.8%
NT-proBNP	1,090 pg/mL
Troponin I	(-)
APTT	26.1 seconds
PT-INR	1.18
Fibrinogen	295 mg/dL
D-dimer	3.1 µg/dL
F_I_O_2_	0.21
pH	7.3
PaCO_2_	49.4 mmHg
PaO_2_	52.7 mmHg
HCO_3_^-^	24.4 mmol/L
Base excess	-2.1
Lactate	27 mg/dL

**Figure 2:**
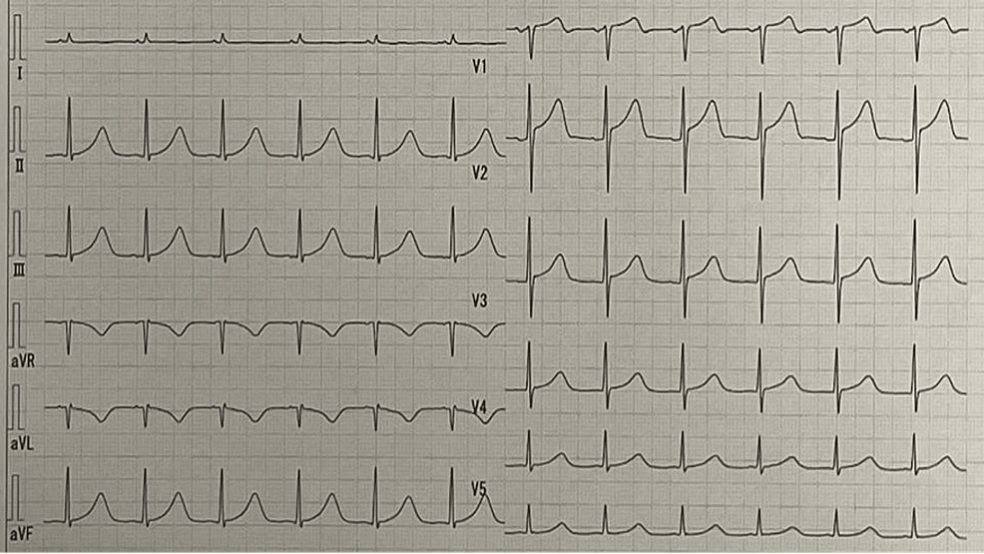
Electrocardiogram under ECMO support. The aVR sign disappeared shortly after ECMO initiation, and hemodynamics stabilized even as ECMO flow was reduced. ECMO = extracorporeal membrane oxygenation

Emergency coronary angiography was planned due to suspected acute coronary syndrome, but immediately after admission to the catheterization laboratory, veno-arterial extracorporeal membrane oxygenation (ECMO) was established due to further hypotension and bradycardia nearly cardiac arrest (heart rate <30 beats/minute). The disappearance of the aVR sign and heart rate recovery were confirmed shortly after the initiation of ECMO support (Figure 2).

**Figure 3:**
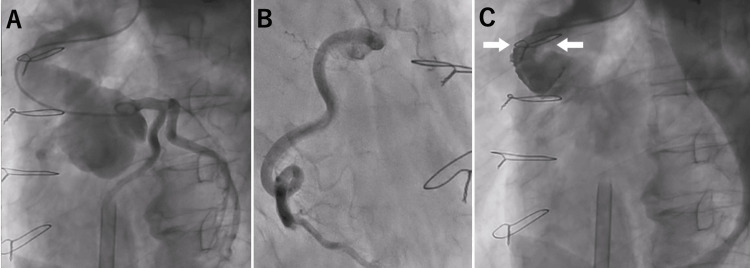
Coronary artery angiography and aortography. Emergency coronary angiography performed under ECMO support revealed no obvious stenosis in the left coronary artery (A) and right coronary artery (B). Aortography showed severe supra-valvular stenosis (arrow) caused by vascular graft kinking (C). ECMO = extracorporeal membrane oxygenation

Coronary angiography performed under ECMO support showed no obvious obstruction in the coronary arteries (Figures 3A, 3B), but aortography showed severe supra-valvular stenosis by vascular graft kinking (Figure 3C).

**Figure 4:**
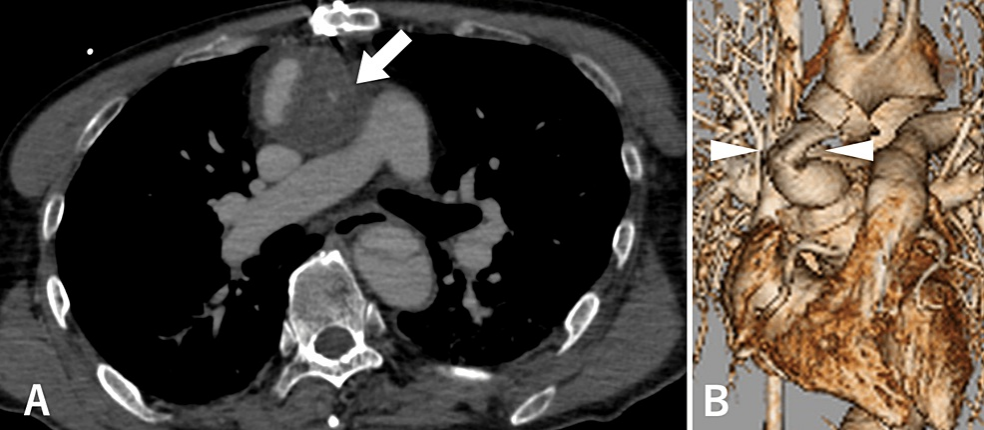
Contrast-enhanced CT scan performed under ECMO. Contrast-enhanced CT scan performed under ECMO support showed extravasation of contrast around the proximal anastomosis site (arrow; A) and the graft kinking induced by hematoma inside the pseudoaneurysm (arrowhead; B). ECMO = extracorporeal membrane oxygenation

Contrast-enhanced CT scan performed under ECMO support showed extravasation of contrast around the proximal anastomosis site and the graft kinking induced by hematoma inside the pseudoaneurysm (Figures 4A, 4B).

While his hemodynamics were maintained even after ECMO flow decreased, the patient was transferred to the operating room under ECMO support for emergency pseudoaneurysm repair to avoid recurrence of instability. His postoperative course was uneventful. The endotracheal tube was removed on the first postoperative day (POD) and he was discharged on the 17th POD. He is doing well at 15 months after repair.

## Discussion

The aVR sign represents diffuse ST-segment depression with ST-segment elevation in aVR on ECG and is considered to indicate a potentially life-threatening disease such as acute coronary syndrome, PE, Takotsubo cardiomyopathy, and severe AS, reflecting extensive subendocardial ischemia [1-4]. We report a case of critical circulatory failure accompanied with the aVR sign caused by severe graft kinking related to acute pseudoaneurysm formation, which is the first case of the aVR sign caused by such a condition to our knowledge.

In the present case, the aVR sign was found along with a fatal condition of nearly cardiac arrest. Following the salvage application of veno-arterial ECMO support, the immediate disappearance of the aVR sign was observed. Aortography and CT scan under ECMO support showed severe graft kinking due to pseudoaneurysm.

Kando et al. suggested that anastomotic pseudoaneurysms could disrupt blood flow by kinking the vascular graft in abdominal aortic aneurysm repair cases [5]. On the other hand, Sakata et al. reported a case of acute Stanford type B aortic dissection in which a sudden increase in left ventricular afterload due to narrowing of the true lumen caused cardiac arrest [6].

These reports indicate that a pseudoaneurysm can cause blood flow disruption similar to an aortic clamp, and a sudden significant increase in left ventricular afterload can cause cardiac arrest by impairing coronary perfusion. In this case, acute supra-valvular aortic stenosis may theoretically present the aVR sign due to rapid left ventricular afterload augmentation causing reduced coronary perfusion and extensive subendocardial ischemia, which is thought to be the same mechanism as severe AS. In this case, the sudden bending of the graft was caused by pseudoaneurysm formation.

Generally, veno-arterial ECMO may increase left ventricular afterload while decreasing left ventricular preload; however, in this case, the application of ECMO support probably worked only to decrease left ventricular preload and left ventricular diastolic pressure owing to the severe graft kinking. The prompt resolution of the aVR sign following ECMO suggests it may have restored coronary perfusion.

The limitation of our speculation is that, ironically, we could not prove the exact pathophysiological hemodynamic parameters because ECMO support was immediately and appropriately applied owing to the fatal state of the patient.

## Conclusions

We experienced a salvaged case of critical circulatory failure accompanied with the aVR sign caused by severe graft kinking related to acute pseudoaneurysm formation. To our knowledge, this is the first report of such a case. The aVR sign is considered a potentially fatal indicator and should be addressed promptly and appropriately. It is also important to consider pathologies such as graft kinking as a cause of the aVR sign.
